# An Intronless *β*-amyrin Synthase Gene is More Efficient in Oleanolic Acid Accumulation than its Paralog in *Gentiana straminea*

**DOI:** 10.1038/srep33364

**Published:** 2016-09-14

**Authors:** Yanling Liu, Zhongjuan Zhao, Zheyong Xue, Long Wang, Yunfei Cai, Peng Wang, Tiandi Wei, Jing Gong, Zhenhua Liu, Juan Li, Shuo Li, Fengning Xiang

**Affiliations:** 1 The Key Laboratory of Plant Cell Engineering and Germplasm Innovation, Ministry of Education, School of Life Sciences, Shandong University, Jinan 250100, China; 2 Department of Information Engineering, Laiwu Vocational and Technical College, Laiwu 271100, China; 3 Key Laboratory for Applied Microbiology of Shandong Province, Ecology Institute of Shandong Academy of Sciences, Jinan 250014, China; 4 Key Laboratory of Plant Molecular Physiology, Institute of Botany, Chinese Academy of Sciences, Nanxincun 20, Fragrant Hill, Beijing 100093, China

## Abstract

Paralogous members of the oxidosqualene cyclase (OSC) family encode a diversity of enzymes that are important in triterpenoid biosynthesis. This report describes the isolation of the *Gentiana straminea* gene *GsAS2* that encodes a *β***-**amyrin synthase (*β*AS) enzyme. Unlike its previously isolated paralog *GsAS1, GsAS2* lacks introns. Its predicted protein product was is a 759 residue polypeptide that shares high homology with other known *β*-amyrin synthases (*β*ASs). Heterologously expressed *GsAS2* generates more *β*-amyrin in yeast than does *GsAS1*. Constitutive over-expression of *GsAS2* resulted in a 5.7 fold increase in oleanolic acid accumulation, while over-expression of *GsAS1* led to a 3 fold increase. Additionally, RNAi-directed suppression of *GsAS2* and *GsAS1* in *G. straminea* decreased oleonolic acid levels by 65.9% and 21% respectively, indicating that *GsAS2* plays a more important role than *GsAS1* in oleanolic acid biosynthesis in *G. straminea*. We uses a docking model to explore the catalytic mechanism of GsAS1/2 and predicted that GsAS2, with its Y560, have higher efficiency than GsAS1 and mutated versions of GsAS2 in *β*-amyrin produce. When the key residue in GsAS2 was mutagenized, it produced about 41.29% and 71.15% less *β*-amyrin than native, while the key residue in GsAS1 was mutagenized to that in GsAS2, the mutant produced 38.02% more *β*-amyrin than native GsAS1.

The Gentianaceae species *Gentiana straminea* Maxim is endemic to western China and the Qinghai-Tibet plateau (4,000 meters above sea level), where it has been exploited as a source of medicinal compounds for at least 2,000 years^1^. Its major pharmaceutically active compounds are secoiridoids and triterpenoids^2^,^3^. Oleanolic acid is one of the most common such triterpenoids. It has anti-hepatitis^4^, antimicrobial^5^, anticancer^6^, and antiapoptotic^6^ activities. Given the restricted extent of growth environments of suitable for *G. straminea* production and limited oleanolic acid yields, the use of metabolic engineering to improve oleanolic acid production in *G. straminea* is an important goal. Many enzymes involved in the biosynthesis of triterpenoids have been the focus of metabolic engineering efforts. The oxidosqualene cyclases (OSCs) are the key enzymes in the cyclization of (3S) 2, 3-oxidosqualene, an important compound in the early steps of triterpenoid biosynthesis^7^,^8^. A number of genes encoding OSCs have been isolated, and their function has been determined via heterologous expression in yeast. *β*-amyrin synthase (*β*AS) genes, which encode key enzymes that catalyze the formation of the precursors of oleanolic acid, have been identified in various plant species^9^,^10^,^11^,^12^,^13^,^14^,^15^,^16^,^17^. The *G. straminea GsAS1* gene was shown to encode a *β*AS^18^, but its function within *G. straminea* has not yet been established.

The majority of eukaryotic genes harbor one or more introns^19^,^20^. However, a significant proportion of plant genes are known to lack introns^21^. Such genes may have originated from intronless ancient genes conserved among archaebacteria, prokaryotes, and eukaryotes, or from RNA-related duplication and retropositionevents^22^,^23^,^24^,^25^. The presence of intronless genes provides an opportunity to evaluate the advantageous that the lack of introns can confer.

Phytosteols and triterpenes are biosynthesized initially via the condensation of acetyl-CoA by the cytosolic mevalonate (MVA) pathway in *G. straminea* (Fig. 1A) and the subsequent cyclization of (3S) 2, 3-oxidosqualene into, respectively, cycloartenol or *β-*amyrin (Fig. 1B). Triterpene cyclization in plants is more complex than sterol synthesis, as the compounds feature a pentacyclic carbon skeleton and a more extensive methyl and hydride shift^26^. In *β-*amyrin biosynthesis, the residues W259 and Y261 of the *Panax ginseng* enzyme PNY are important for the formation of the D/E-ring of *β-*amyrin^27^,^28^, and the product-determining role of the DCTAE motif has recently been demonstrated by applying a site-directed mutagenesis approach to the *Euphorbia tirucalli* gene that encodes *β-*amyrin synthase (EtAS)^29^. Subtle structural and hence electrostatic differences at the active site of an enzyme can radically alter its substrate and product specificity, and can also affects its catalytic efficiency^30^. To date, only a few studies have reported analyses of the catalytic functions of particular residues in the active site of OSCs, perhaps owing to a lack of suitable docking models of the enzyme and intermediate compounds.

Most terpenoids are produced *in planta* in very small amounts, so their commercial exploitation is often hampered by uneconomic yields^31^. As our understanding of terpeniod synthesis and metabolic engineering of plants improves, new opportunities to enhance productivity and alter terpenoid product profiles are becoming available^32^,^33^. Higher product yield and recovery for compounds including carotenoids, artemisinin, patchoulol, and linalool/nerolidol have been achieved in various organisms including *E. coli, Arabidopsis thaliana, Artemisia annua*, tobacco, potato, and maize^34^,^35^,^36^,^37^,^38^,^39^,^40^,^41^. Since *β*-amyrin is a key precursor of the triterpenoid saponin pathway, over-expression of *βAS* may possibly result in increased saponin production. Expressing a *β*-amyrin synthase gene from *Panax japonicus*had in rice increased the accumulation ofoleanolic acid^42^.

No intronless OSC genes have been reported in plants^43^. Here, we describe the isolation of an intronless *G. straminea* OSC sequence (*GsAS2*). We compared its product profile with that of its intron-containing paralog *GsAS1*. Heterologously expressed *GsAS2* generates relatively more *β*-amyrin in yeast than does *GsAS1*, and constitutive expression of *GsAS2* in *G. straminea* results in an up to 5.7 fold increased in oleanolic acid yield compared to wild type plants. And a comparison between *GsAS1* and *GsAS2* showed that the latter’s product is the more important for the production of oleanolic acid in transgenic plants of *G. straminea*. We performed substrate and enzyme docking modeling and found that one key residue may ultimately explain the observed differences in oleanolic acid accumulation between GsAS2 than GsAS1. Site-directed mutagenesis and heterologous expression in yeast cells demonstrated that, relative to GsAS1 and the H560 and F560 mutant forms of GsAS2, the GsAS2 produced *β*-amyrin more efficiently.

## Results

### An intronless OSC sequence identified from *G. straminea*

**Figure 2:**
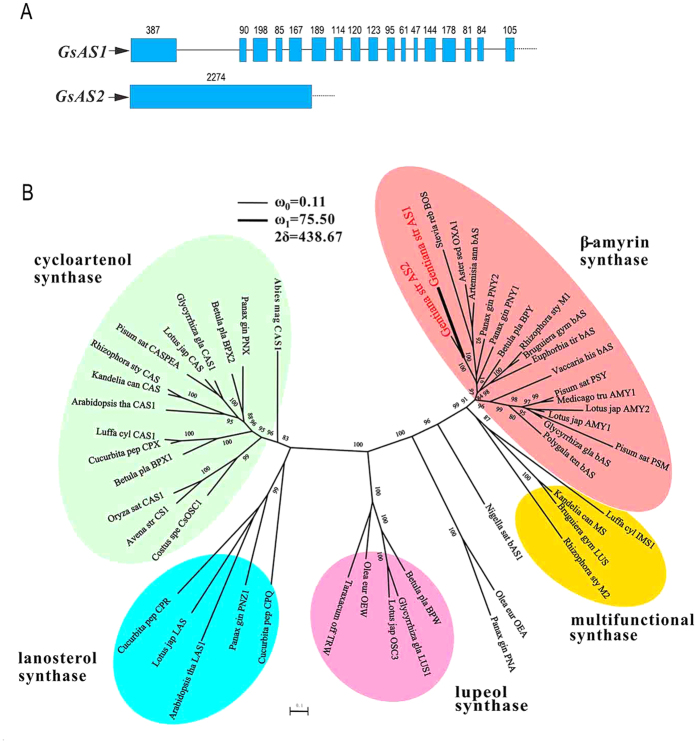
The structure and phylogeny of *GsAS1* and *2*. (**A**) The structure of the genomic loci for *GsAS1* and *2*. (**B**) A maximum likelihood tree based on codon alignment, annotated with relevant bootstrap values. The tree was estimated using the GTR + Γ + I substitution model implemented within PhyML software. The scale bar indicates the number of substitution sites.

A full length *β*AS cDNA sequence, named *GsAS1*, has already been isolated from *G. straminea*^18^. The 920 bp amplicon produced by degenerate PCR was shown by sequencing to comprise two distinct sequences, one of which was *GsAS1*. The other, when used to generate a full length sequence, was named *GsAS2* (KJ467352); its length was 2,277 bp, encoding a predicted 759 residue polypeptide containing one DCTAE and four QW motifs (Supplementary Fig. S1A). The nucleotide sequence of the genomic locus for *GsAS2* was identical to that of the full length cDNA (Supplementary Fig. S1B). Thus, *GsAS2* is an intronless *OSC* sequence. However, 16 introns are present in the genomic sequence of *GsAS1* (Fig. 2A). Phylogenetic analysis showed that *GsAS2* resembled other OSCs, with the closest matches being *GsAS1* and *β*AS enocoding genes present in *Aster* sp. and *Artemisia* sp. (Fig. 2B). The GsAS2 peptide sequence is highly homologous to that of GsAS1 (81.1% identity) and to those of the *β*ASs present in *Panax ginseng* (AB014057, 76.2%), *Aster sedifolius* (AY836006, 73.1%), *Glycyrrhiza glabra* (AB037203, 73.1%), *Betula platyphylla* (AB055512, 74.2%), and *Euphorbia tirucalli* (AB206469, 74.3%).

The ω values associated with the *GsAS1* and *GsAS2* sequences were estimated for each branch using a free ratio model. Since ω represents the non-synonymous to synonymous substitution ratio, the indication was that *GsAS2* has evolved under purifying selection (ω = 0.06), whereas a higher ω value (0.25) was evident for the branch leading to *GsAS1* (Supplementary Table S1). The ω value per codon in the background (ω_0_) and foreground (ω_1_) lineages were, respectively, 0.11 and 75.50 (2ΔL = 434.74, P < 0.01), as estimated using branch site model A (Supplementary Table S1). *GsAS1* appears to have evolved recently, at an accelerated rate,under positive selection.

### Transcription of *GsAS2* and *GsAS1* in *G. straminea*

**Figure 3:**
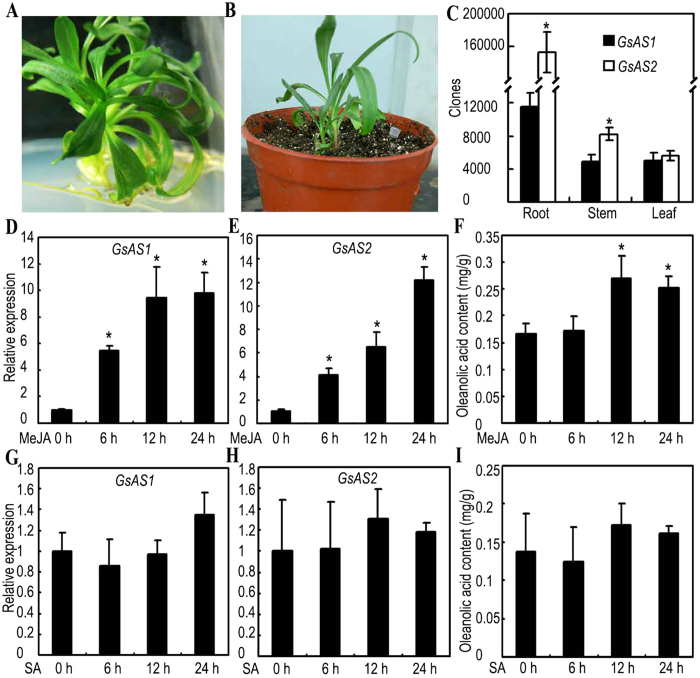
Transcription profiles of *GsAS1* and *GsAS2* and oleanolic acid content in *G. straminea*. (**A,B**) *G. straminea* plants used in this study. (**C**) The transcription of *GsAS1* and *GsAS2* in the root, stem, and leaf as assessed via qRT-PCR. The *β-actin* gene from *G. straminea* was used as the reference gene for quantification. The bars indicate the standard error (n = 3), *Statistically significant differences as analyzed by Student’s *t*-tests and ANOVA test (P ≤ 0.05). (**D,E,G,H**) The transcription of *GsAS1* and *GsAS2* in *G. straminea* plants treated with MeJA (**D,E**) and SA (**G,H**). The bars indicate the standard error (n = 3). *Statistically significant differences as analyzed by Student’s *t*-tests and ANOVA test (P ≤ 0.05). (**F,I**) Oleanolic acid content in *G. straminea* plants treated with MeJA (**F**) and SA (**I**). The bars indicate the standard error (n = 10). *Statistically significant differences as analyzed by Student’s *t*-tests and ANOVA test (P ≤ 0.05).

The transcription profiles of *GsAS1* and *GsAS2* were analyzed in different organs of *G. straminea* plants (Fig. 3A,B). The standard curves calculated for both *GsAS1* and *GsAS2* were very similar to one another (Supplementary Fig. S2). Both *GsAS1* and *GsAS2* were highly expressed in roots. *GsAS1* transcript levels in roots were 2.3 fold greater than the levels in either the leaves or stems. *GsAS2* transcript levels in roots was 27.5 fold greater than that in leaves and 18.6 fold greater than in stems. *GsAS2* expression was significantly higher than *GsAS1* in roots (13.3 fold increase) and in stems (1.6 fold increase). No difference in the transcript levels of *GsAS1* and *GsAS2* was observed in leaves (Fig. 3C).

*GsAS1* and *GsAS2* transcription was strongly induced by methyl jasmonate (MeJA) treatment, producing, after 24 h, respectively, 9.8 and 12.2 fold increases over the level of mock-treated plants (Fig. 3D,E). The oleanolic acid content of the plants treatment with MeJA was also increased significantly (Fig. 3F). Salicylic acid (SA) did not affect the transcription level of *GsAS1* or *GsAS2* and did not affect oleanolic acid content (Fig. 3G–I).

### *GsAS2* Encodes a functional *β*AS with more contribution to oleanolic acid accumulation than *GsAS1*

**Figure 4:**
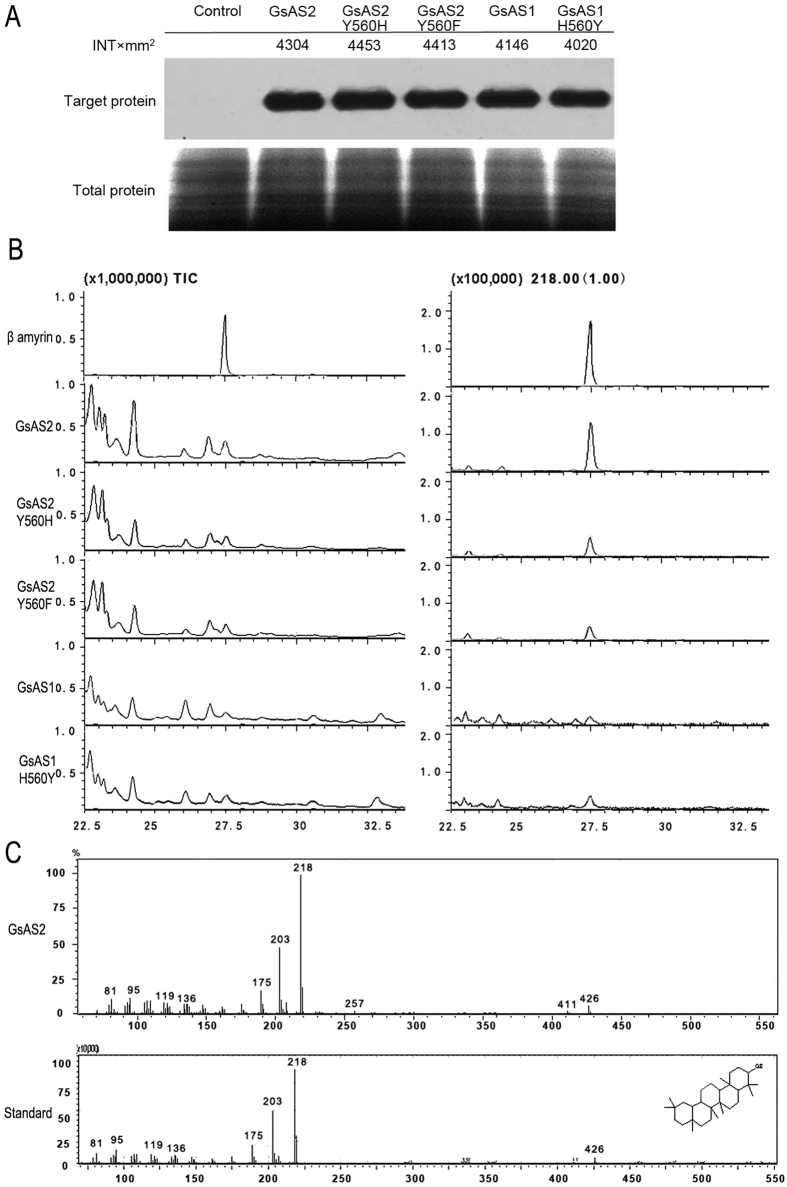
*GsAS2* encodes a functional *β*AS. (**A**) The expression of the wild type and mutant forms of GsAS1 and GsAS2, as assayed by western blotting. Control: protein extracted from yeast cells harboring an empty vector. Target protein: western blot profiles. Total protein: SDS-PAGE profile of total protein stained with Coomassie blue R250. (**B**) GC chromatographs. The trace displays the output from yeast cells carrying either an empty vector, *GsAS1, GsAS1-H560Y, GsAS2, GsAS2-Y560H,*or *GsAS2-Y560F*. (**C**) Mass spectra of the standard (*β*-amyrin) and the output of yeast cells harboring *GsAS2*.

Heterologous expression of *GsAS1* in yeast has shown that it encodes a functional *β*AS^18^. When the *GsAS2* open reading frame was placed under the control of the methanol-inducible *AOX1* promoter within the *pPICZA* expression vector and introduced into *Picha pastoris,* the GsAS2 protein was detected (Fig. 4A) and it generated a product with the same retention time (27.50 min) as a *β-*amyrin reference standard. Control cells (carrying only an empty vector) did not express this product (Fig. 4B). The mass spectrum for this enzymatic product was consistent with that of *β*-amyrin (Fig. 4C). These results suggest that the enzyme encoded by *GsAS2* cyclizes oxidosqualene to form *β*-amyrin and thus suggest that *GsAS2*, like *GsAS1*, is a functional *βAS* gene. Comparison of the product accumulation in the assays with the heterologously expressed forms of GsAS1 and GsAS2 showed that the yield of *β*-amyrin by GsAS2 was about 12.9 times greater than that of GsAS1 (Table 1) suggesting that *GsAS2* has a higher potential for oleanolic acid accumulation than *GsAS1* when heterologously expressed in yeast.

**Figure 5:**
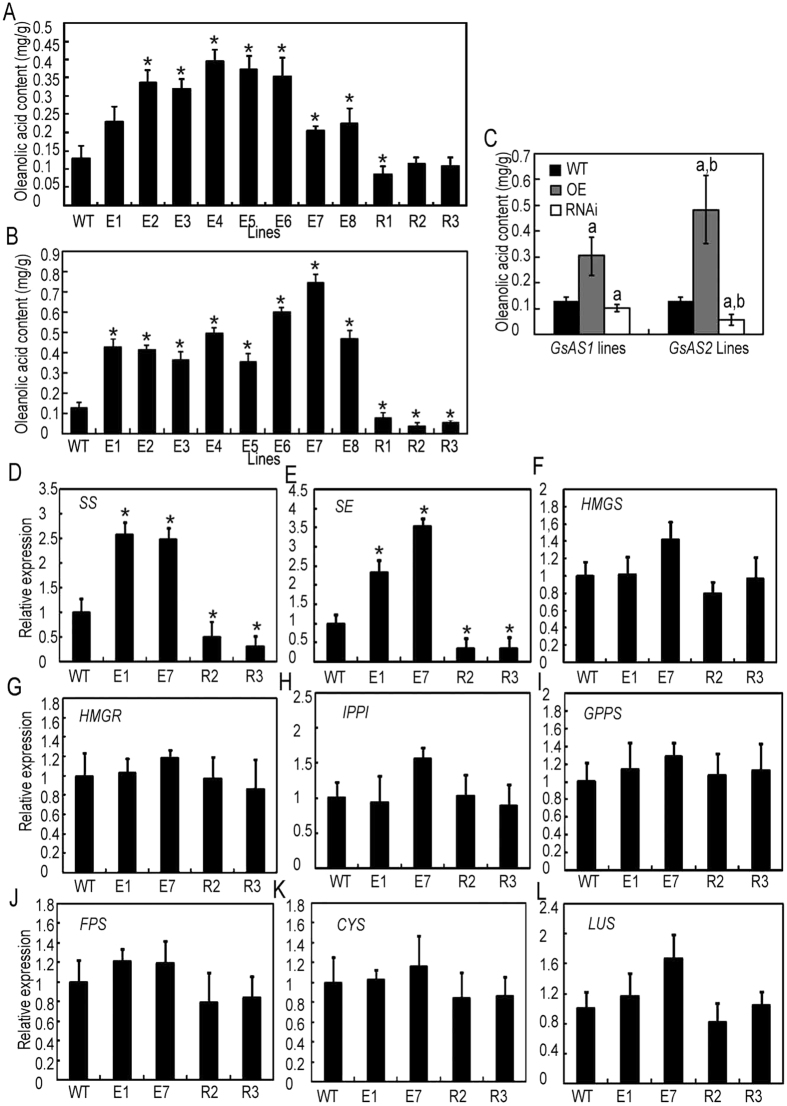
GsAS2 apparently produces oleanolic acid more efficiently than *GsAS1*. (**A,B**) Oleanolic acid content following the up- or down-regulation of *GsAS1*(**A**) and *GsAS2*(**B**). WT: wild type, E: over-expression lines, R: RNAi lines. The bars represent the standard error (n = 10). *Statistically significant differences which were analyzed by Student’s *t*-tests and ANOVA (P ≤ 0.05). (**C**) The average increase or decrease (fold) in oleanolic acid content in transgenic plants with *GsAS1* or *2* over-expression and RNAi. The bars represent the standard error (*n* = 3). a: Statistical significance between means was inferred by a *t*-test compared with wild type (P ≤ 0.05), b: Statistical significance between *GsAS1* and *GsAS2* transgenic plants samples means evaluated with Student’s *t-*test compared. (WT: wild type, E: over-expression lines, R: RNAi lines). (**D–L**) The effect of *GsAS2* over-expresion on transcription of triterpene biosynthesis pathway genes in *GsAS2* transgenic plants (WT: wild type, E: over-expression lines, R: RNAi lines).

The relative enzymatic activity of *GsAS1* and *GsAS2* was also assessed in terms of oleanolic acid accumulation in the *GsAS1* and *GsAS2* over-expression transgenic *G. straminea* lines and in *GsAS1* and *GsAS2* RNAi suppression *G. straminea* lines (Fig. 5A–C, Supplementary Fig. S3). Quantitative RT-PCR (qRT-PCR) analysis showed that the transcript abudance for both *GsAS1* and *GsAS2* was higher in the over-expression lines than in the wild type and that the transcript abudance for both *GsAS1* and *GsAS2* was lower in the RNAi lines than in the wild type (Supplementary Fig. S4A,B). Based on the transcript abundance results, particular over-expression transgenic lines of *G. straminea* were chosen for southern blot analysis. The expression levels rose as the number of transgene copies increased in transgenic *G. straminea.* The *GsAS2* transgenic *G. straminea* line with the highest *GsAS2* expression level had 4 copies of the transgene in its genome (Supplementary Fig. S4C). HPLC analysis showed that the oleanolic acid content of the *GsAS2* over-expression lines on average 3.7 (between 1.6 and 5.7) fold higher than that of the wild type, while the *GsAS1* over-expression lines, oleanolic acid accumulation was 2.3 fold higher that of the wild type (the range was 1.8–3.0 fold) (Fig. 5C). Oleanolic acid content was reduced by 65.9% in the *GsAS2* RNAi plants but was reduced by just 21.0% in the *GsAS1* RNAi plants (Fig. 5C). These results indicated that *GsAS2* was also more efficient than *GsAS1* in directing oleanolic acid accumulation in *planta*.

When the transcription levels of other genes involved in the triterpene pathway were assessed in *GsAS2* over-expression (E1 and E7) and RNAi (R2 and R3) lines, the abundance of *SS* and *SE* transcripts was found to be similar to that of *GsAS2*. For *SS*, the fold difference compared to the WT was 2.6 in E1 and 2.5 fold in E7, while the difference was 0.5 fold in R2 and 0.3 fold in R3 (Fig. 5D). Similarly, as compared to the WT, the abundance of *SE* transcripts in the E1, E7 and the R2 and R3 lines was, respectively, 2.3 and 3.5 fold higher and 0.4 and 0.3 fold lower (Fig. 5E). No differences was observed in the expression of other genes of the triterpene pathway, including *HMGS, HMGR, IPPI, GPPS, FPS, CYS*, or *LUS* (Fig. 5F–L).

### The possible mechanism for the apparent increased catalytic efficiency of GsAS2 over GsAS1 for *β*-amyrin synthesis

**Figure 6:**
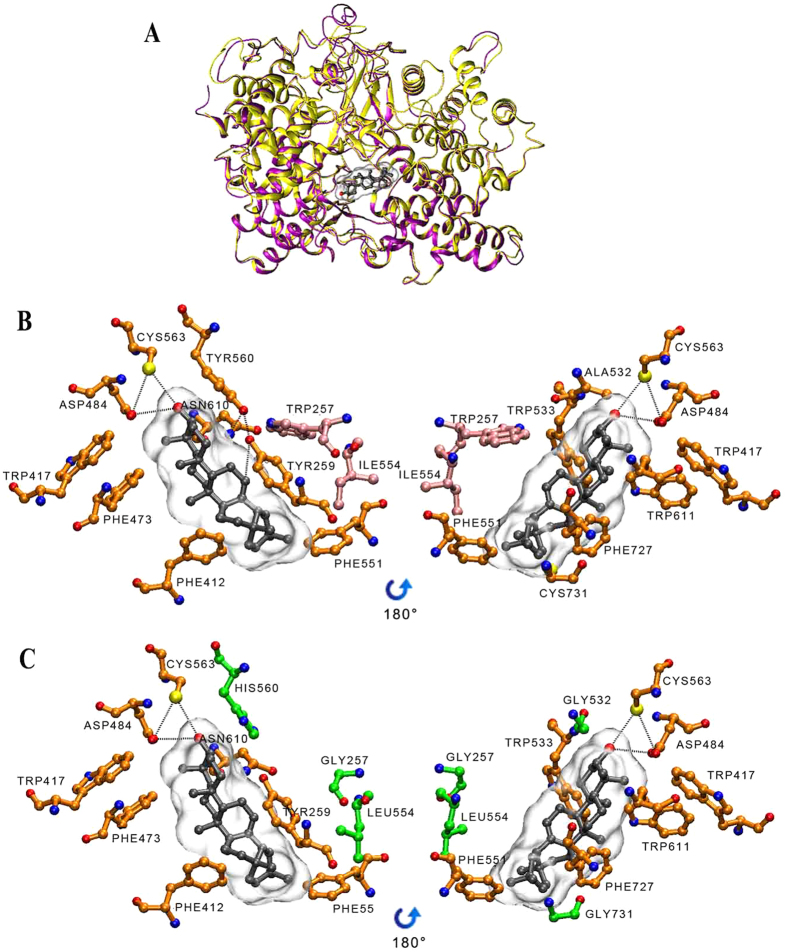
Modelling of GsAS1 and GsAS2 in complex with *β*-amyrin. (**A**) Ribbon diagram representation of GsAS1 (yellow) and GsAS2 (purple). The *β*-amyrin molecule (black) positioned in the active site. (**B**) Substrate combination model of GsAS2. (**C**) Substrate combination model of GsAS1. Residues interacting with *β*-amyrin are indicated by orange colored bonds. Different residues between GsAS1 and GsAS2 interacting with *β*-amyrin are indicated by green colored bonds. Residues interacting indirectly with *β*-amyrin are indicated by pink colored bonds. The hydrogen bonds are shown as dashed lines.

To explore possible mechanisms for the apparent functional improvement of GsAS2 over GsAS1 in the production of *β*-amyrin, we superimposed I-TASSER derived models of GsAS1 and GsAS2 with a backbone root mean square deviation of 2.09 Å (Fig. 6A), indicating their very high overall structural likeness. This was as expected, given their similar enzymatic activity.

Both enzymes harbored two (α/α) barrel domains connected by loops, as well as three smaller *β* structures (Fig. 6A). After docking with an oleanylcation, both the GsAS1 and GsAS2 active site cavities became surrounded by hydrophobic residues, except for their polar caps (Fig. 6B,C). The D484 residue is free to form a hydrogen bond with the substrate’s 3-hydroxy group, while C563 can serve as a hydrogen-bonding partner (Fig. 6B,C). Their intermediate product oleanylcations were surrounded with the aromatic residues Y259, Y412, W417, F473, W533, F551, Y560, W611, and F727 (Fig. 6B,C). The active sites of GsAS1 and GsAS2 differed with respect to H/Y560,G/W257, L/I554, G/C731, G/A532 (Fig. 6B,C). In GsAS2, the W257 indole ring lay close to (about 3 Å) the Y259 benzene ring, bringing Y259 close to C12, C13, and C18 of the oleanylcation (respectively, 2.3, 3.5, and 3.2 Å). The Y560 hydroxy group was able to interact with that of Y259 (4.5 Å) and the amido group of N610 (4.1 Å). However, in GsAS1, G257 was unable to interact with Y259, while the H560 imidazole group lay further away from the Y259 hydroxy group (>7 Å) - although it was still able to interact with N610 (3.9 Å) (Fig. 6B,C).

The docking results identified two substitutions (H560Y and G257W) as potentially responsible for the apparent differences in the *β-*amyrin production of GsAS1 and GsAS2. The behavior of W257 has been reported by Kushiro *et al*.^28^, while the functional effect of the residue 560 has not been investigated. Previously, it has been shown that GsAS1 produces *β*-amyrin^18^, suggesting that both the H and Y560 variant may be functional with respect to *β-*amyrin synthesis. In this study, the two GsAS2 mutants Y560H and Y560F and GsAS1 H560Y were generated to investigate the effect of the H/Y560 residue on *β-*amyrin production and the catalytic properties of the enzymes. Each sample was divided into two equal portions. When one portion was processed by western blotting, the bands representing the mutant and native enzymes had very similar densities (Fig. 4A), implying a similar titer of these enzymes forms in yeast. The other sample portion was used to test triterpenoid biosynthesis in yeast extraction and analysis with GC-MS. The highest *β-*amyrin levels were measured in yeast cells harboring GsAS2, the relative amounts of *β-*amyrin in yeast cells harboring GsAS1, mGsAS1-H560Y, mGsAS2-Y560H, and -Y560F compared to cells with GsAS2, were, respectively, 7.77 ± 0.64%, 10.72 ± 0.51%, 58.71 ± 3.42%, 28.85 ± 2.30% (Table 1 and Fig. 4B). The two mutated GsAS2 sequences produced, respectively, 41.29% and 71.15% less *β*-amyrin than the native GsAS2, while the GsAS1 mutant produced 38.02% more *β*-amyrin than native GsAS1 (Table 1). These results suggest that the Y560 residue in the GsAS2 active site confers higher efficiency in *β*-amyrin formation than the H560 residue in the active site of GsAS1, and this difference may explain the higher efficiency of *GsAS2* on oleanolic acid accumulation compared to *GsAS1*.

## Discussion

The terpenoids form one of the largest classes of plant secondary metabolites, and the *OSC* genes represent an important component of their synthesis. Characterization of *OSC* function has been achieved indirectly via heterologous expression in yeast for genes isolated from ginseng^13^ and pea^15^, and directly in the model species *A. thaliana*^44^,^45^. To date, the contribution of these genes to terpenoid (especially oleanolic acid) synthesis has not been well-elucidated in a medicinal plant species. We show here that the over-expression of *GsAS2* enhanced the accumulation of oleanolic acid, a finding consistent with our previous results showing that oleanolic acid accumulation is positively related to the expression level of *GsASs*^46^. The relevant transgenic *G. straminea* plants could therefore make a significant contribution towards the engineering of this species to produce economically-viable amounts of this valuable metabolite.

Most of the *OSC* gene sequences described to date feature one or more introns, reaching as many as 17 in *AsbAS1* (from oat) and 16 in *AtCAMS1* (from *A. thaliana*)^13^,^47^. Unlike *GsAS2* which is lacks introns (Supplementary Fig. S1), the previously characterized *G. straminea* gene *GsAS1* features 16 introns (Fig. 2A) and shows similar exon patterns to other OSC genes (data not shown). *GsAS1* might represent the gene directly derived from the ancestor gene with a conserved structure, while the intronless *GsAS2* represents more recent newer evolutionary origin. It has been suggested that a likely origin of intronless genes is retroposition^48^. The expression of a retroposed gene copies is expected not to correlate with its source gene, because it obtains new regulatory elements from its site of insertion^48^. Therefore, new function often occurs with the daughter gene (retrogene), rather than with a parental gene. However, a daughter gene may inherit promoters and enhancer elements from their parental genes, resulting in a daughter gene with regulatory properties similar to the parental gene^43^. Thus, both daughter gene and parental genes have the possibility to undergo neofunctionalization. There is a report of this type of case; the daughter gene actually maintained the ancestral function, while the partental gene underwent neofunctionalization^49^. Here, *GsAS2* appeared to be more strongly transcribed than *GsAS1* (Fig. 3C), with the result that alterations (either up or down) in the former’s transcription level had a greater effect on the plant’s oleanolic acid content than did the latter’s (Fig. 5A–C and Supplementary Fig. S3). An evolutionary analysis of the *GsAS2* sequence suggested that its function is likely to have been conserved as a result of purifying selection (Supplementary Table S1). However, *GsAS1* appears to have evolved at an accelerated rate through positive selection; it may either still be in the process of neo-functionalization or may represent a multi-functional triterpene cyclase that generates an undetected alternative product. The observation that GsAS1 makes a lesser contribution to the synthesis of oleanolic acid than GsAS2 supports the notion that *OSC* genes multiply via gene duplication, with positive selection driving one copy to neo-functionalize via a process of non-synonymous mutations, while the other retains the original function^17^.

Subtle morphological and electrostatic differences at the active site of an enzyme can radically alter their enzymatic capacities^30^. Here, the active sites of GsAS1 and GsAS2 differed with respect to H/Y560, G/W257, L/I554, G/C731, and G/A532. Among these, the G552, L554, and G731 residues in GsAS1 have similar chemical and physical characteristics with A552, I554 and C731 in GsAS2 (Fig. 6B,C), and may therefore contribute little to the *β*-amyrin synthesis activity. Substitutions (H560Y and G257W) between GsAS1 and 2 affected *β*-amyrin production. Here, in GsAS2, the Y560 hydroxyl group lay close to Y259, together with N610 forming a hydrophilic environment surrounding C13 of the oleanylcation, which ostensibly improves a deprotonation from C13 for the formation of *β-*amyrin (Fig. 6B). The GsAS1 H560 residue has a smaller side chain than that of Y560, and so may interact less strongly with Y259 in the context of deprotonation (Fig. 6C). The mutagenesis of GsAS1 and GsAS2 showed that the presence of H560 was associated with reduced *β*-amyrin production relative to the protein form with Y560 (Table 1). Additionally, in previous research, residue W259 in PNY (corresponding to the W257 in GsAS2) was demonstrated to play a key role in *β*-amyrin synthesis^25^, suggesting that W257 may also play a role in *β*-amyrin synthesis. Therefore GsAS2 was more effective than GsAS1 in *β-*amyrin production in yeasts, which was consistent with the results of greater accumulation of oleanolic acid upon constitutive expression of *GsAS1/2*.

Up-regulating a rate-limiting enzyme gene within a given metabolic pathway represents an effective means of raising productivity^31^. A typical example relates to the accumulation in *Eleutherococcus senticosus* of phytosterols and saponins via the heterologous expression of a SS-encoding gene isolated from ginseng, in which the levels of product were increased by between 2.0 and 2.5 fold^50^. Similarly, the artemisinin content in *Artemisia annua* plants over-expressing *FPS* exhibited a 2.5 fold increase compared to the non-transgenic control^51^. In the present experiment, however, it proved possible, via the over-expression of *GsAS2,* to enhance oleanolic acid content by nearly 6 fold (Fig. 5B).

When secondary metabolic flux is disturbed or changed by over-expression or reduced expression of genes, the expression level of other genes in the pathway can be up- or down- regulated by feedback or feed-forward effects with the products of the target genes. Over-expression of *Panax ginseng* squalene synthase (PgSS1) in adventitious roots of transgenic *P. ginseng* was followed by the up-regulation of genes of triterpene synthesis such as *βAS*, and this resulted in a remarkable increase in ginsenoside content^52^. However, there is little direct evidence for the regulatory function of *βAS* genes in the biosynthesis of triterpene saponins. Here, we found that the expression of *SS* and *SE* were up-regulated in plants when *GsAS2* was over-expressed (Fig. 5D,E), showing that the level of *GsAS2* expression affects the transcription of certain genes upstream in the triterpene pathway. The increased oleanolic acid content resulting from the over-expression of *GsAS2* shows that this gene is an important component of the triterpenoid synthesis pathway in *G. straminea.*

In conclusion, *GsAS2* exhibit more important roles on oleanolic acid accumulation than *GsAS1* in *G. straminea* at several aspects. The transcription abundance of the *GsAS2* is higher than that of *GsAS1* especially in roots which have the highest oleanolic acid content. Furthermore, this function was further emphasized by lower accumulation of oleanolic acid in *GsAS2* RNAi lines compared to *GsAS1* RNAi lines (Fig. 5C). For apparent enzyme efficiency, GsAS2 accumulated 12.9 fold more *β*-amyrin that did GsAS1 when expressed heterologously in yeast (Table 1). The important residues (Y560) were identified by adocking model and mutation studies showed that the form of GsAS2 with Y560 had accumulated more oleanolic acid than both GsAS1 and various mutated forms of GsAS2. All of these results highlight the remarkable function of the intronless *GsAS2* and lay a foundation for the use of this gene improving the triterpenoid production via metabolic engineering.

## Materials and Methods

### Plant materials and tissue culture

Embryogenic calli of *G. straminea* were generated from sterilized seeds cultured at 25 ± 1 °C on MS basal medium^53^ supplemented with 30 g/L sucrose and 2 mg/L 2, 4-dichlorophenoxyacetic acid. Calli were subcultured every two weeks. Regeneration was induced by transfer to differentiation medium (IB medium), which was MS medium supplemented with 0.5 mg/mL indole acetic acid and 0.5 mg/mL 6-benzyl adenine.

### Gene isolation

Total RNA of *G. straminea* was extracted and reverse transcribed as described by Liu *et al*.^18^. Two degenerate primers (5′-TGGCTTTCGATA(T)CTTGGA-3′ and 5′-CCACCG(A)TTTTTG(A)CTCTGTA-3′) were designed based on highly conserved regions of reported OSCs genes from other plants including *P. ginseng, Betula platyphylla, Aster sedifolius,* and *Euphorbia tirucalli*. RT-PCR was then performed using the first strand cDNA of *G. straminea* as a template. The resulting 920 bp RT-PCR amplicon was cloned into the pMD18-T vector (Takara, Japan), transformed into *E. coli* DH5α cells, and sequenced. Overlapping 5′ and 3′ sequences were obtained using RACE PCR, based on a Full RACE Core Set (Takara, Japan) and the primer pair 5′-CTCAACCCAACAAGCAAGC-3′ and 5′-ATGGGTTGCAGAAGATGG-3′. A set of ten independent 5′ and 3′ clones was sequenced, and their sequences were aligned to obtain a consensus sequence^15^. For full length gene isolation, the same primer pairs of *GsAS2* were amplified with the total DNA of the *G. straminea* as a template. The primers for *GsAS1* are listed in Supplementary Table S2.

### OSC sequence phylogeny

A multiple alignment of OSC sequences was performed using Muscle 3.6 software^54^ with some manual editing. Proteins sequence alignment was transformed into codon sequences with the help of theaa2 DNA script. Maximum likelihood phylogenies were constructed from the codon sequence alignment using PHYML software^55^ based on the GTR + Γ + I substitution model. The free ratio model of CODEML, implemented within the PAML4 software package^56^, was used to estimate the lineage-specificity of the non-synonymous to synonymous substitution ratio ω. A branch site analysis, which compared the nearly neutral model with Model A^56^, was performed to test the assumption that the foreground ω value of a specific branch was >1 at sites where positive selection appeared to have acted within a specific lineage. The resulting likelihood ratio tests were performed at the 5% level.

### Transcription profiles of GsAS1 and GsAS2

The roots, stems, and leaves of regenerated plants in IB medium were separated, and RNA was extracted by using TRIzol reagent (Invitrogen, USA) following the manufacturer’s instructions. Then RNA was spectrophotometrically quantified and reversed transcribed into cDNA, which subsequently used as a template for qRT-PCR to analyze the expression of *GsAS1* and *GsAS2* in different tissues. The qRT-PCR primers targeted *GsAS1* and *-2*; their sequences were: GsAS1-F/-R: 5′-TCCTCTGATTATATGCTTGT/5′-ACCATCCTCATTCTGAT and GsAS2-F /-R: 5′-GGAGGATTAGCAGCATCT/5′-CCATCTTGTCGTTGTGAAT. The qRT-PCR analysis used a SYBR Green I real-time PCR detection system (Applied Biosystems, USA), using the *β-actin* gene from *G. straminea* as the reference gene. Each 15 μL reaction contained 0.2 μM of each primer and 2 μL of a 1:10 dilution of the prepared first-strand cDNA. The thermalcycling program was as follows: a denaturation step of 95 °C/2min, followed by 40 cycles of 95 °C/10s, 58 °C/20s, 72 °C/20s. A melting curve analysis was performed over the range 80–95 °C at 0.5 °C intervals. An absolute quantification of *GsAS1* and *-2* transcript abundance was obtained through the use of a standard curvethat plotted logarithm of initial copies of template DNA against the threshold cycle number from a serial dilution of the 3.337 × 10^10^ copies/μL of a *pPICZA-GsAS1-GsAS2* plasmid that contained atandemly linked sequences of *GsAS1* and *2*.

The plants treated with 50 μM MeJA or 50 μM SA for 0 h, 6 h, 12 h, and 24 h were harvested and frozen with liquid nitrogen. The treated materials were used to analyze the expression of *GsAS1* and *GsAS2* or the content of oleanolic acid. The treated regenerated plants used to analyze the content of oleanolic acid were air-dried, ground to a powder, suspended in ten volumes of methanol, and exposed to 60 min of ultrasonic homogenization. The material was then centrifuged at 10,000 g for 10 min, and the supernatant was filtered through a 0.45 μm membrane. 10 μL extracts of each sample was injected into an HPLC instrument with an Ultrasphere C18 column (150 mm × 4.6 mm i.d., Phenomenex). Methanol::water (9::1) was used as the mobile phase, the flow-rate was maintained at 0.8 mL·min^−1^, and the effluent was monitored at 209 nm. The peak area of oleanolic acid was integrated by a Classvp5.0 system, using oleanolic acid as an external standard; a standard calibration curve was generated from data measured for a range of concentrations (20–1000 mg·mL^−1^).

### Functional analysis via heterologous expression of OSC enzymes in yeast

*P. pastoris* strain X33, which was cultured in YPD medium (1% yeast extract, 2% peptone and 2% glucose, supplemented with 100 μg/mL Zeocin^TM^ (Invitrogen, USA)), was used in this study. *GsAS2* with *Kpn*I and *Xho*I restriction enzyme sites was amplified using the forward primer: 5′-ATCGGTACCATGTGGAGGCTAAAGATTGCA-3′ and the reverse primer: 5′-CGGCTCGAGCAGATGGCAATGGCACTCTCT-3′. *GsAS1* with *EcoR*I and *Xho*I restriction enzyme sites was amplified from the *pPICZA*-*GsAS1* plasmid using the forward primer: 5′-ACTACTAGTGAATTCATGTGGAGGCT-3′ and the reverse primer: 5′-CGGCTCGAGCGTCCGGCACTTGCTTGCGGT-3′^15^. The reverse primers were designed to delete the stop codons. Both fragments contained full ORFs and were subcloned into the *P. pastoris* expression vector *pPICZA* (Invitrogen, USA) under the control of the methanol-inducible promoter, *5*′*AOX1*. The PICZA-*GsAS1*,*2* and empty vector *pPICZA* were then transformed separately into *P. pastoris* strain X33 using electroporation according to the manufacturer’s instructions.

The extraction of membrane proteins was achieved using a Mem-PER Eukaryotic Membrane Protein Extraction Reagent Kit (Thermo, USA) following the manufacturers protocol. For western blotting, 1 volume of 2 × SDS sample buffer was added to the protein extract and the mixture was incubated at 70 °C for 10 min, followed by a centrifugation at 12,000 g for 10 min. The resulting supernatant was subjected to SDS–PAGE (10% polyacrylamide) to resolve the protein species present, then transferred to a PVDF (polyvinylidene difluoride) membrane (EMD Millipore, USA) using a semi-dry electrophoretic transfer cell (Bio-Rad, USA). The subsequent immunodetection assay was based on an anti-His primary antibody (Abcam, UK) and an HRP-conjugated anti-rabbit IgG secondary antibody (CST, USA). The hybridized membrane was immersed in freshly prepared HRP reaction solution (Advansta, USA) for 1–2 min, and then exposed to X-ray film for 1 min. Yeast cells harboring *GsAS2* and empty vectors were cultured at 30 °C to OD_600_ = 2~6 in minimal glycerol medium (MGY, 1.34% yeast nitrogen base (YNB), 1% glycerol, 4 × 10^−5^% biotin). The cells were collected, resuspended in minimal methanol medium (MM, 1.34%YNB, 4 × 10^−5^% biotin, 0.5% methanol) to OD_600_ = 1.0, and incubated at 30 °C for 4 d by adding 100% methanol to a final concentration of 0.5% every 24 h. The incubated yeast cells were finally collected and disrupted with 2 mL of 20%KOH/50% EtOH (1/1, v/v) for every 25 mL MM medium. The products were extracted twice with 2 mL hexane. The extracts and 1–3 μg standards *β*-amyrin (Sigma, USA) were analyzed by using a GCMS-QP2010 GC/MS System fitted with an Agilent DB-5MS column (29.5 m × 250 μm internal diameter, 0.25 μm film) (JW Scientific, USA). The inlet, transfer line, and ion source temperatures were set at, respectively, 270 °C, 270 °C and 200 °C, and the oven temperature was programmed to 200 °C for 2 min, raised to 270 °C at 10 °C per min, and held at 270 °C for 30 min. The flow rate of the carrier helium was 1.5 mL per min. Splitless injections (8 μL) were used, and mass spectral data in the *m/z* range 70–550 were acquired. A standard calibration curve for *β*-amyrin was generated from data measured for a range of concentrations (10–50 mg·mL^−1^).

### Vector construction and transformation of *G. straminea*

The plant over-expression vector *pK7WG2D* and the RNAi vector *pK7GWIWG2D(II)* (Invitrogen, USA), both constructed using the Gateway technique, were combined with two independent *GsASs* cDNA sequences and transformed into seven day old *G. straminea* calli using particle bombardment^57^. After a 24 h period in darkness, the calli were transferred to selective medium containing 50 mg/L kanamycin. Surviving calli was tested for the presence of the *GsASs* with a PCR assay targeting the CaMV 35S promoter sequence (primer sequences: 5′-GCAGAGGCATCTTCAACG-3′ and 5′-TTCGATCATGGGCAGAAGACGAC-3′).

### Characterization of regenerated transgenic plants

Surviving calli were transferred to the aforementioned regeneration medium and plants able to be successfully regenerate were evaluated for the presence of the transgenic constructs with a PCR assay targeting the CaMV 35S promoter sequence, as above. The number of copies of the transgene in plants was assessed by Southern blotting. The transcription levels of genes involved in triterpene synthesis was monitored by qRT-PCR. Oleanolic acid content was analyzed by HPLC, as mentioned detailed above.

### Three dimensional modeling and mutagenesis

Three dimensional models of the GsAS1 and GsAS2 proteins were generated using I-TASSER v2.1 software^58^. The resulting models had C-scores 1.30 (GsAS1) and 1.37 (GsAS2). AutoDock v4.2 software^59^ was used to visualize the GsAS1/2/*β*-amyrin complex. The three dimensional structure of *β*-amyrin was obtained from http://zinc.docking.org (ID 3978269). In the data preprocess before docking, the structure was restrained within a grid box (40 × 86 × 60 points in each dimension) that covered the GsAS2 binding pocket. The identity of the binding pocket was inferred assuming similarity with the crystal structure of the OSC-lanosterol complex (PDB code: 1W6K). Docking searches were performed using the Lamarckian genetic algorithm, with a maximum of 25,000,000 energy evaluations and other options set as the default. Ten potential models were returned, which were then ranked on the basis of binding energy. The top ranked model was assumed to be the most likely. The models were graphically rendered using VMD software (http://www.ks.uiuc.edu/Research/vmd)^60^.

The H560Y substitution in GsAS1/2 was chosen for site mutagenesis experiment. The mutant was generated using a PCR-based strategy to mutate the Y in GsAS2 to H and F (Phe), as well as H to Y in GsAS1. The mutagenesis sites were designed into primers^61^, and the PCR strategy was followed that of Edelheit *et al*.^62^ The mutation primers were listed in Table 2.

### Statistical analysis

Results arepresented as the means of three independent biological replicates in all statistical tests. Inferential statistical tests were implemented in SPSS17.0 software and included one-way ANOVA followed by Duncan’s multiple range tests and Student’s *t*-tests (see figure legends for specific tests for particular experiments). P values ≤0.05 were interpreted as indicating statistically significant differences.

## Additional Information

**How to cite this article**: Liu, Y. *et al*. An Intronless *β*-amyrin Synthase Gene is More Efficient in Oleanolic Acid Accumulation than its Paralog in *Gentiana straminea. Sci. Rep.* **6**, 33364; doi: 10.1038/srep33364 (2016).

## Supplementary Material

Supplementary Information

## Figures and Tables

**Figure 1:**
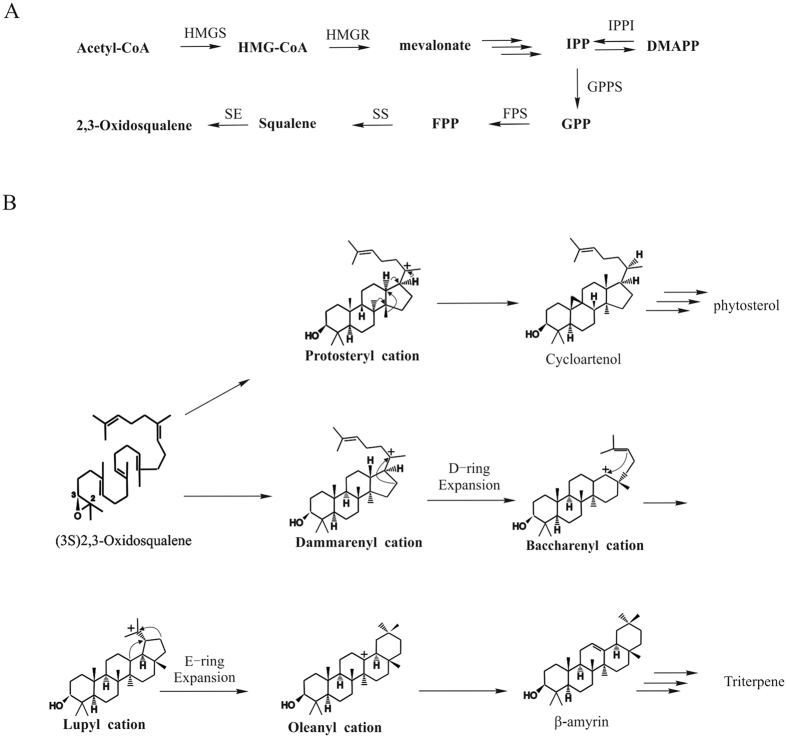
(**A**) Early steps in biosynthesis of phytosterols and triterpenoid saponins leading to the common precursor 2, 3-oxidosqualene. HMG-CoA: 3-hydroxy-3-methylglutaryl-CoA, IPP: Isopentenyl pyrophosphate, DMPP: dimethylallyl pyrophosphate, GPP: geranyl pyrophosphate, FPP: farnesyl pyrophosphate. HMGS: 3-hydroxy-3-methylglutaryl-CoA synthase, HMGR: 3-hydroxy-3-methylglutaryl-CoA reductase, IPPI: IPP isomerase, GPPS: geranyl diphosphate synthase, FPS: farnesyl diphosphate synthase, SS: squalene synthase, SE: squalene epoxidase. (**B**) The biosynthetic pathway of (3S)-2, 3-oxidosqualene into phytosteol andtriterpene via cycloartenol and *β*-amyrin.

**Table 1:** Detected quantities of *β*-amyrin generated by the native and mutant forms of GsAS1 and GsAS2.

	Amount of *β-*amyrin in the product of yeast cells relative to that of GsAS2 (%)	Amount of *β-*amyrin in the product of yeast cells relative to that of GsAS1 (%)
GsAS2	100.00 ± 10.11^a^	
mGsAS2-Y560H	58.71 ± 3.42^b^	
mGsAS2-Y560F	28.85 ± 2.30^c^	
GsAS1	7.77 ± 0.64^d^	100.00 ± 14.18
mGsAS1-H560Y	10.72 ± 0.51^d^	138.02 ± 11.41*

Values are presented as means ± standard error (*n = *3). Values followed by the same letter within a column do not differ significantly (P ≤ 0.05) according ANOVA. *Significant difference (P < 0.05) between GsAS1 wild type and its mutant was determined by a *t*-test means.

**Table 2:** Point mutation primers of *GsAS1* and *GsAS2*.

		Primer
GsAS1	Sense	GAGCATGAGTATGCCGAGTGTAC
H560Y	Anti-sense	ACTCGGCATACTCATGCTCAAT
GsAS2	Sense	AGCATGAGCATGTTGAATGTACTGCATC
Y560H	Anti-sense	CATTCAACATGCTCATGCTCAATCACAAT
GsAS2	Sense	AGCATGAGTTTGTTGAATGTACTGCATC
Y560F	Anti-sense	CATTCAACAAACTCATGCTCAATCACAAT
