# Supplementary material for: An Intronless β-amyrin Synthase Gene is More Efficient in Oleanolic Acid Accumulation than its Paralog in Gentiana straminea
Source: Sci Rep. 2016 Sep 14;6:33364. doi: 10.1038/srep33364 (PMC5022052; doi:10.1038/srep33364)
Supplement: Supplementary Information [file srep33364-s1.doc]

**An Intronless *β*-amyrin Synthase Gene is More Efficient in Oleanolic Acid Accumulation than its Paralog in *Gentiana straminea***

Yanling Liu^1,2,*^, Zhongjuan Zhao^1,3,*^,Zheyong Xue^4,*^, Long Wang^1,*^, Yunfei Cai^1^, Peng Wang^1^, Tiandi Wei^1^, Jing Gong^1^, Zhenhua Liu^1^, Juan Li^1^, Shuo Li^1^and Fengning Xiang^1^

^1^The Key Laboratory of Plant Cell Engineering and Germplasm Innovation, Ministry of Education, School of Life Sciences, Shandong University, Jinan 250100, China;

^2^Department of Information Engineering, Laiwu Vocational and Technical College, Laiwu 271100, China;

^3^Key Laboratory for Applied Microbiology of Shandong Province, Biology Institute of Shandong Academy of Sciences, Jinan 250014, China;

^4^Key Laboratory of Plant Molecular Physiology, Institute of Botany, Chinese Academy of Sciences, Nanxincun 20, Fragrant Hill, Beijing 100093, China.

^*^These authors contributed equally to this work

Correspondence and requests for materials should be addressed to F.X. (email:

[xfn0990@sdu.edu.cn](mailto:xfn0990@sdu.edu.cn))

**Supplementary Table S1.** Likelihood ratio test for *GsAS1* and *GsAS2.*

| model | Branch | Estimates of Parameters^a^ | -InL | 2ΔL^b^ | P |
| --- | --- | --- | --- | --- | --- |
| M0  (one ratio) |  | 0.11614 | -62159.44 |  |  |
| M1  (free ratio) |  | ω_GsAS2_=0.2474,  ω_GsAS2_=0.0554 | -61826.26 | 666.36 | <0.001 |
| M1a  (near neutral) |  | *p*_0_=0.9993, *p*_1_=0.00007  ω_0_=0.11172,  ω_1_=1 | -61902.95 |  |  |
| Ma  (Branch site) | *GsAS1* | *p*_0_=0.91808, *p*_1_=0.07273, *p*_2_=0.00918,  ω_0_=0.11031,  ω_1_=1,  ω_2_=75.49988 | -61683.62 | 434.74 | <0.001 |
|  | *GsAS2* | *p*_0_=0.92631, *p*_1_=0.07369, *p*_2_=0,  ω_0_=0.11050,  ω_1_=1,  ω_2_=1 | -61685.58 |  |  |

^a^ The proportion of sites (*p*_0_, *p*_1_, ....) estimated to have ω values ω_0_, ω_1_, ....

^b^ 2ΔL = twice the log-likelihood difference between Ma and M1a.

**Supplementary Table S2.** Primers for full length gene isolation of *GsAS1*

|  |  | primer |
| --- | --- | --- |
| GsAS1-1  GsAS1-1 | Sense  Anti-sense | ATGTGGAGGCTAAAAATCGGTG  CACTGCTGGTATGCTCTGCTTG |
| GsAS1-2  GsAS1-2 | Sense  Anti-sense | CAGCTTTGCAGTCTTCCGACG  CGGAGAATTTCGTGTGTATGC |
| GsAS1-3  GsAS1-3  GsAS1-4  GsAS1-4  GsAS1-5  GsAS1-5  GsAS1-6  GsAS1-6  GsAS1-7  GsAS1-7  GsAS1-8  GsAS1-8  GsAS1-9  GsAS1-9  GsAS1-10  GsAS1-10 | Sense  Anti-sense  Sense  Anti-sense  Sense  Anti-sense  Sense  Anti-sense  Sense  Anti-sense  Sense  Anti-sense  Sense  Anti-sense  Sense  Anti-sense | GGCCATCTGAACAATATATTTC  CTTCTCCGAGTATTCTCATGC  GGATTCTTGACCATGGTAGTGT  GGTGTAATTCGACAAACAAATC  GTTACACTGTGAGCCATATAATC  TGTTTCATTGTGGCATCTAGAGC  GTCGCTATATCACCGTTGGATC  CCTTTGGACATGTGACGGTACATGC  GACTTTCTCAGATCAAGATCATG  GAATAAAACAAGAGCGTCGATTGC  GAGATTGAGAGTTTCATCAC  CGGCCTTACGAACAACTTGGC  GATGGTGGTTGGGGAGAAAGC  GATTAACAGTTTTGCTGCACAA  GTCCATACTGCTTGGGCTATG  CGGCACTTGCTTGCGGTATTC |


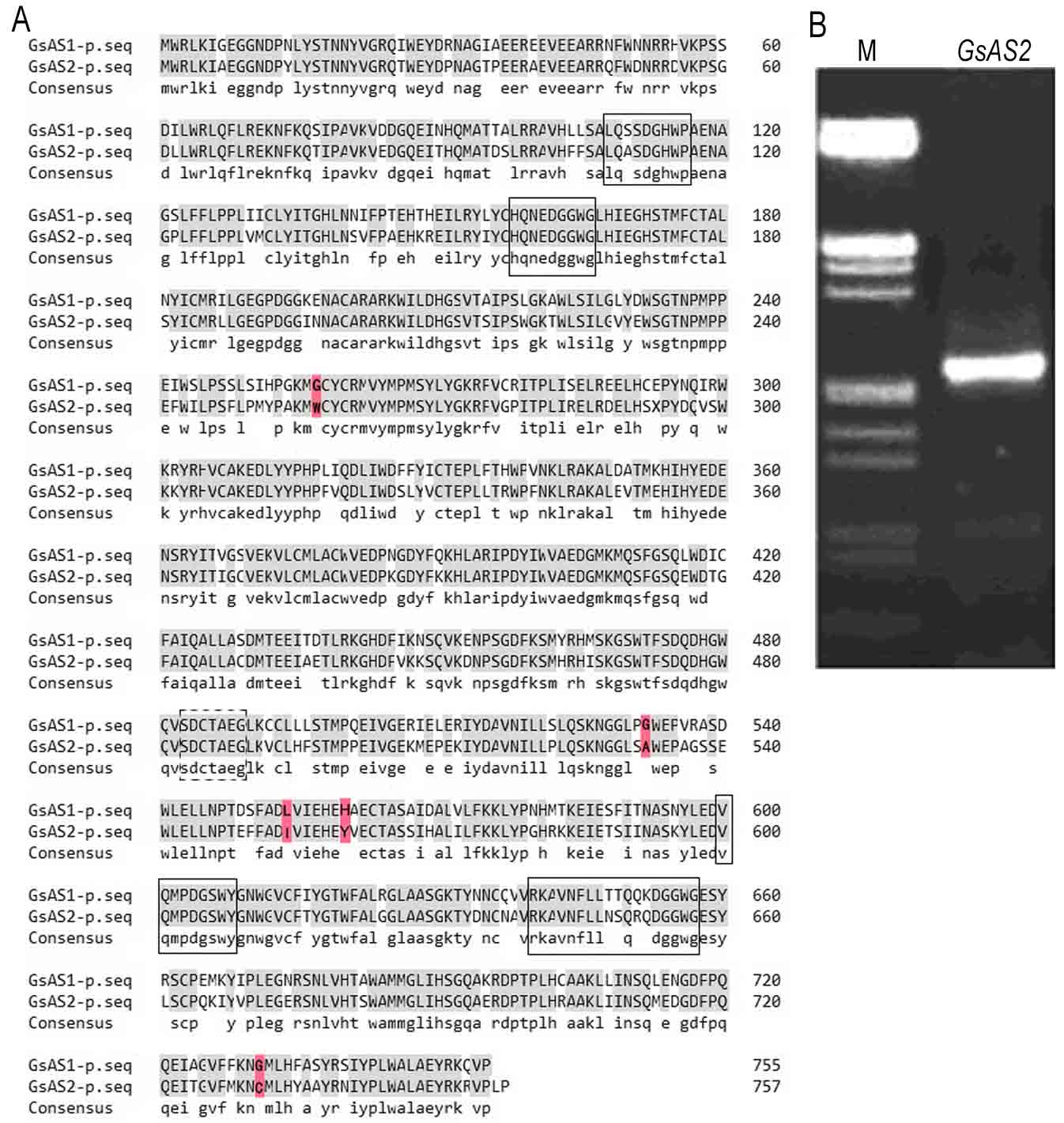


**Supplementary Figure S1.** (A) Peptide alignment of GsAS1 and GsAS2.The two polypeptides share an 81.5% level of identity (615/755). Their QW and DCTAS motifs are boxed by the full line and the dotted line, respectively. Different residues between GsAS1 and GsAS2 that putatively interact with *β*-amyrin in their substrate pocket have a red background. (B) The full length clone of *GsAS2* obtained from *G. straminea* genome.


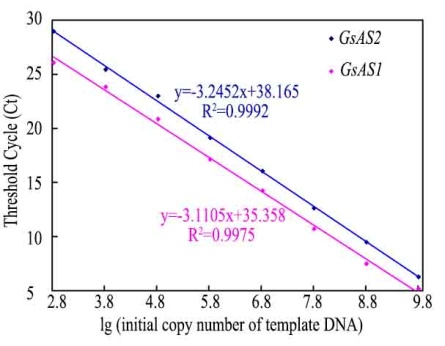


**Supplementary Figure S2.** Standard curves used to quantify *GsAS1* and*-2* amplicons. The standard curve was established by plotting logarithmic initial copies of template DNA against the threshold cycle number from a serial dilution of the *pPICZA-GsAS1-GsAS2* plasmid, which contains tandemly-linked *GsAS1* and *2* sequences.


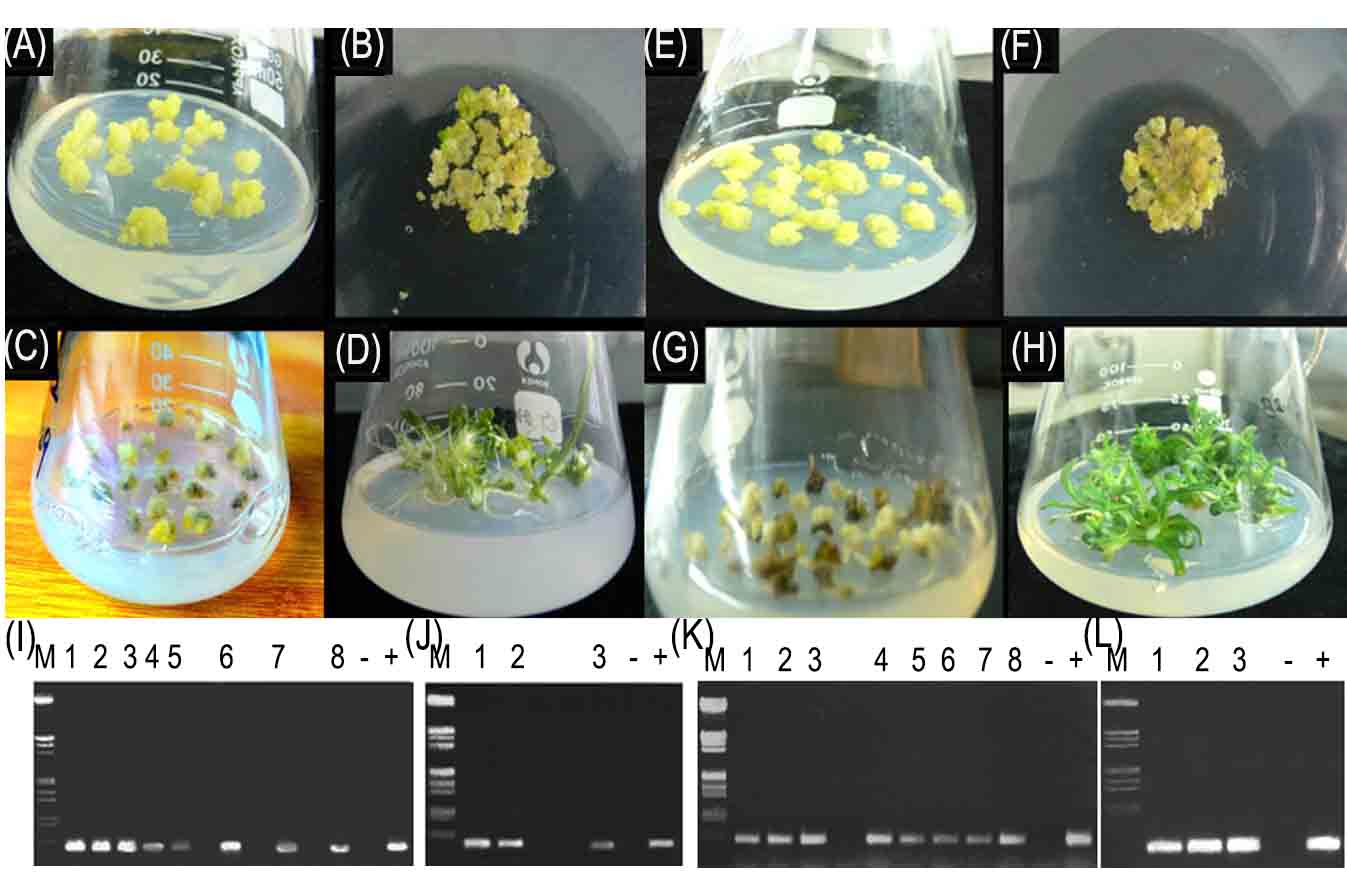


**Supplementary Figure S3.** Transgenic *G. straminea* plants which over-expressed or suppression expressed *GsAS1*/*2* were obtained. (A, E) Calli prior to transformation. (B, F) Calli after transformation. (C, G) Transformed calli after selection on kanamycin containing medium. (D, H) Regenerated transgenic plants. (I-L) PCR validation of transgenic status, based on genomic DNA extracted from plants putatively carrying pK7WG2D-*GsAS1* or *2* (I, K) or pK7GWIWG2D-*GsAS1* or *2* (J, L), M: DNA size marker.


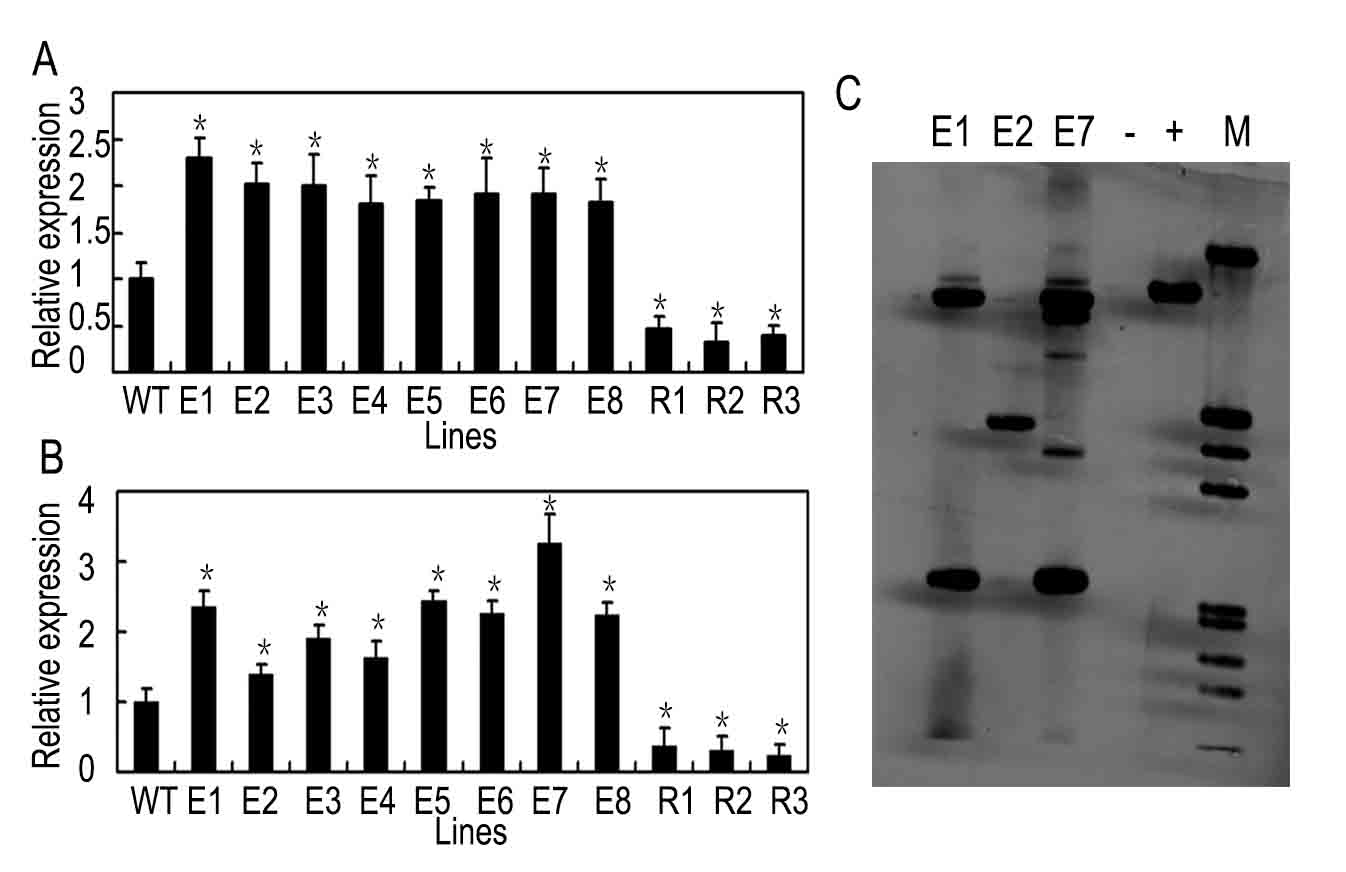


**Supplementary Figure S4.** (A, B) *GsAS1* or *2* transcript abundance in transgenic plants as assessed using qRT-PCR, WT: wild type, E: over-expression lines, R: RNAi lines. The bars represent the standard error of the mean (n=3). *, Statistically significant differences analyzed by Students’s *t*-test (P ≤ 0.05). (C) Southern blot of several *GsAS2* over-expression transgenic lines. The *GsAS2* over-expression transgenic lines E1, E2, and E7 were chosen for southern blot analysis.
